# Cholinesterase based amperometric biosensors for assay of anticholinergic compounds

**DOI:** 10.2478/v10102-009-0011-5

**Published:** 2009-06

**Authors:** Miroslav Pohanka

**Affiliations:** Center of Advanced Studies, Faculty of Military Health Sciences, University of Defence, Hradec Kralove, Czech Republic

**Keywords:** biosensor, acetylcholinesterase, butyrylcholinesterase, amperometry, organophophates, organophosphonates, carbamates

## Abstract

Biosensors are analytical devices being approachable for multiple analytes assay. Here, biosensors with intercepted acetylcholinesterase (AChE) or butyrylcholinesterase (BChE) are presented as tool for assay of anticholinergic compounds such as pesticides, nerve agents and some natural toxins. Principle of assay is based on evaluation of cholinesterase activity and its pertinent decrease in presence of analyte. Nerve agents, pesticides, anticholinergic drugs useable for treatment of Alzheimer′s disease as well as myasthenia gravis and aflatoxins are enlisted as compounds simply analyzable by cholinesterase biosensors.

## Introduction

Inhibition of two enzymes in body: acetylcholinesterase (AChE; EC 3.1.1.7) and butyrylcholinesterase (BChE; EC 3.1.1.8) is a common toxicology pathway of many compounds. Nerve agents, insecticides such as organophosphates as well as carbamates and some drugs may be named (Pohanka *et al*., 2009a). The function of BChE is not crucial. On the other hand, AChE is an important enzyme in body. AChE is bound in outer membrane of neurons and terminates neurotransmission through neural cleft by hydrolyzing acetylcholine (Soreq and Seidman, 2001). Another action of AChE is the cholinergic anti-inflammatory pathway associated with vagus nerve (Pavlov *et al*., 2003).

Intoxication by organophosphates, organophosphonates and carbamates is manifested by bronchospasm, bradycardia, miosis, lacrymation and diarrhoea. Furthermore, confusion, coma and/or respiratory failure caused by overstimulation of nicotinic and muscarinic receptors would be also observed when anticholinergic compound is administered (Eddieston *et al*., 2008).

The inhibition of cholinesterases *in vitro* was found approachable for construction of analytical devices such as biosensors. The cholinesterase based biosensors are very effective tools for assaying of anticholinergic compounds. Even, estimation of *in vitro* impact of some drugs or presence of other anticholinergic compound is accompanied by inhibition of cholinesterases used in biosensor (Pohanka *et al*., 2008a, b). The present review is aimed at summarization of the most important facts about cholinesterase based biosensors. Assay of typical anticholinergic compounds such as organophosphates, organophosphonates and carbamates by biosensors as well as biosensors principles and constructions are mentioned in the review.

## The most important cholinesterase inhibitors

There are available many different compounds with anticholinergic properties. Some of them, e.g. aflatoxins, are natural toxins with strong inhibition of AChE but not BChE (Egbunike and Ikegwuonu, 1984; Cometa et al., 2005; Pohanka et al., 2008e). Drugs (e.g. tacrine and galantamine being administered for treatment of Alzheimer′s disease cognitive manifestation and myasthenia gravis are also modulators of AChE activity (Musial *et al*., 2007). Some studies even found several plant secondary metabolites as strong inhibitors of cholinesterases (Lopez *et al*., 2002).

**Figure 1:**
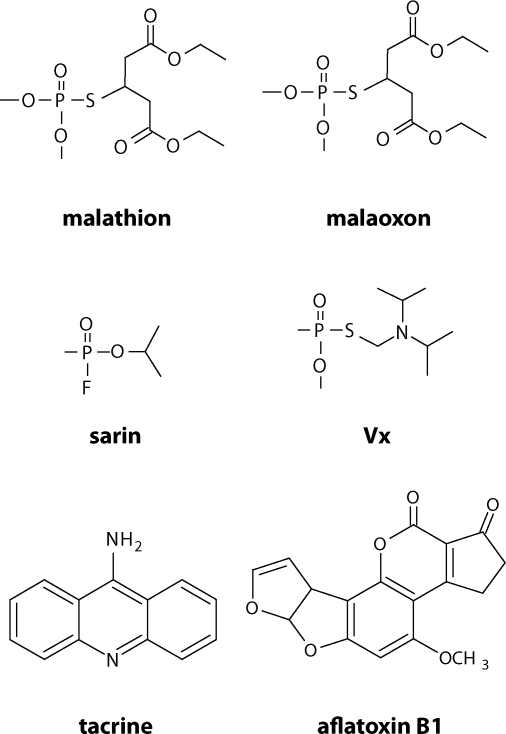
Examples of some anticholinergic compounds.

Typical artificial inhibitors of cholinesterases are organophosphates and organophosphonates. These two groups of anticholinergic compounds are irreversible inhibitors of both AChE and BChE. Toxic organophosphates are predominantly used as pesticides. Paraoxon, parathion, diisopropylfluorphosphate (DFP), malaoxon, malathion, chlorpyrifos and dichlorvos may be listed as typical examples (Marrs, 1993). Toxicity of some pesticides is attenuated by replacing of oxygen phosphate residuum by sulfur. It is e.g. derivate paraoxon and parathion or malaoxon and malathion. Toxicity is recovered due to mixed function oxidases in insect body. Warm blooded have not enzymatic apparatus for this pathway and thioderivates are slowly activated using cytochrome P-450 (Buratti *et al*., 2005). Some of organophosphonates were found useable as nerve agents in military points of view. Sarin, soman, tabun, cyclosarin and VX are well known representatives (Barthold *et al*., 2005). Examples of some above mentioned compounds are attached in Figure 1.

## Amperometric assay

Amperometric biosensors are a good alternative to the potentiometric ones. Amperometric biosensors provide linear output signal dependence on concentration of analyte in comparison with the logarithmic relationship typical for potentiometric sensors (Ghindilis *et al*., 1998). Amperometric biosensors are quite simple in comparison with potentiometric devices. A simple noble metal wire can serve as the physicochemical transducer. Carbon paste electrodes are well known and also the screen-printed electrodes became one of the adopted possibilities. Carbon paste electrodes proved to have limited reproducibility due to coarsely defined carbon suspensions parameters. The best results were historically obtained with the screen-printed electrodes. The screen-printed electrodes can be simply produced on a mass scale. Miniaturization of electrodes seems to be ideal for flow-through based techniques (Pijanowska *et al*., 2003). Amperometric measurement can be realized in either two- or three-electrode configurations. The two electrode system is simpler. It consists of the reference and working electrodes. The two electrode configuration could be preferred for systems where currents or better current densities are low. If the value of current is high, the instability of reference electrode potential could occur and the three electrode configuration should be used. In this case, current flows between auxiliary and working electrodes.

The current going through the working electrode can be defined according to the Faraday law as follows:

$$i=\frac{Q}{t}=\frac{mFz}{Mwt}$$

The meaning of symbols is common; *i* means electric current, which is proportional to the electric charge *Q* transmitted during time interval *t*. The Faraday constant *F* is approximately equal to 96,485 C/mol. The transformed ions are described by molecular weight (*Mw*) and charge *z*; total mass of substances, which either precipitated or dissolved on the electrode, is expressed as *m*. An indifferent ion should be added to solution for polarization suppression.

## Amperometric evaluation of cholinesterase activity using biosensor

Biosensors are analytical devices consisting from biorecognition element and a proper sensor element (Brecht and Gauglitz, 1995). BChE and mainly AChE are promising recognition elements for biosensors construction (Pohanka *et al*., 2008b). Evaluation of cholinesterase activity is the crucial factor in the construction of biosensors. Though the acetylcholine is commercially available, the reaction is not simply detectable. Typically pH electrodes would be applied as sensor detecting acidification of medium by releasing of acetic acid. Nowadays, glass electrodes are replaced by the semiconductors such as ion sensitive field effect transistor (ISFET) and light addressable potentiometric sensors (LAPS) being more approachable (Arkhypova *et al*., 2001; Yoshinobu *et al*., 2004). The replacement of potentiometric by amperometric principle has been found appropriate.

There is possibility to evaluate activity of cholinesterase by an amperometric principle in two ways. The first is based on performance of cholinesterase commonly with cholineoxidase (ChOx; EC 1.1.3.17) and oxygen or hydrogen peroxide amperometric transducer (Campanella *et al*., 2007). ChOx oxidizes creating choline up betaine. A biosensor based on AChE and ChOx was found sensitive for assay of some pesticides such as pirimiphosmethyl in levels demanded by Europe Union (Del Carlo *et al*., 2005).

**Scheme 1:**
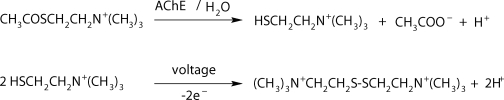
Amperometric evaluation of AChE using acetylthiocholine

The second way being frequently performed for amperometric evaluation of AChE activity is based on replacement of native substrate acetylcholine by an alternative acetylthiocholine. The mechanism is lucidly depicted in Scheme 1. Electrochemical oxidation of reaction product thiocholine is started by applied voltage (Pohanka *et al*., 2009b). Biosensors with intercepted AChE working on above mentioned principle are promising devices for multiple assays. Pesticide paraoxon (Pohanka *et al*., 2008c), dichlorvos (Valdes-Ramirez *et al*., 2008), sulfotep (Kandimalla and Ju, 2006), natural toxic compound aflatoxin (Pohanka *et al*., 2008e), nerve agents (Pohanka *et al*., 2009b) and current as well as novel anticholinergic drugs (Pohanka et al., 2008d) may be mentioned as examples of typical analytes.

## Conclusions

Amperometric biosensors with intercepted cholinesterases are promising tools for evaluation of many anticholinergic compounds. Assay of pesticides, nerve agents and aflatoxins are typical analytes. The second way of biosensor performance is evaluation of drugs administered to patients suffering from Alzheimer′s disease and myasthenia gravis.
